# Estimating the sensorimotor components of cybersickness

**DOI:** 10.1152/jn.00477.2018

**Published:** 2018-07-25

**Authors:** Séamas Weech, Jessy Parokaran Varghese, Michael Barnett-Cowan

**Affiliations:** Department of Kinesiology, University of Waterloo, Waterloo, Ontario, Canada

**Keywords:** balance control, perception, self-motion, vection, virtual reality

## Abstract

The user base of the virtual reality (VR) medium is growing, and many of these users will experience cybersickness. Accounting for the vast interindividual variability in cybersickness forms a pivotal step in solving the issue. Most studies of cybersickness focus on a single factor (e.g., balance, sex, or vection), while other contributors are overlooked. Here, we characterize the complex relationship between cybersickness and several measures of sensorimotor processing. In a single session, we conducted a battery of tests of balance control, vection responses, and vestibular sensitivity to self-motion. Following this, we measured cybersickness after VR exposure. We constructed a principal components regression model using the measures of sensorimotor processing. The model significantly predicted 37% of the variability in cybersickness measures, with 16% of this variance being accounted for by a principal component that represented balance control measures. The strongest predictor was participants’ sway path length during vection, which was inversely related to cybersickness [*r*(28) = −0.53, *P* = 0.002] and uniquely accounted for 7.5% of the variance in cybersickness scores across participants. Vection strength reports and measures of vestibular sensitivity were not significant predictors of cybersickness. We discuss the possible role of sensory reweighting in cybersickness that is suggested by these results, and we identify other factors that may account for the remaining variance in cybersickness. The results reiterate that the relationship between balance control and cybersickness is anything but straightforward.

**NEW & NOTEWORTHY** The advent of consumer virtual reality provides a pressing need for interventions that combat sickness in simulated environments (cybersickness). This research builds on multiple theories of cybersickness etiology to develop a predictive model that distinguishes between individuals who are/are not likely to experience cybersickness. In the future this approach can be adapted to provide virtual reality users with curated content recommendations based on more efficient measurements of sensorimotor processing.

## INTRODUCTION

Virtual reality (VR) technology allows a user to experience a simulated environment through an array of sensory stimulation apparatuses (Hale and Stanney 2014). Such arrays typically consist of electronic visual displays and sound devices, which can be updated in real time based on the input of manual controllers, inertial motion units, and (depending on the hardware) eye tracking. Costs for these components have fallen in recent years, leading to the rapid adoption of VR hardware by enthusiasts. Although the technology has a wealth of potential in a variety of settings, such as industrial skills training, consumer entertainment, and clinical rehabilitation, the sickness and discomfort experienced by many users of VR limit further adoption (Biocca 1992; Keshavarz and Hecht 2011; Kim et al. 2005). Sickness during VR exposure, termed “cybersickness” (CS), has been studied in some detail in recent years. The phenomenon is related to several maladies under the general term “motion sickness,” including car sickness or seasickness (Riccio and Stoffregen 1991), visually induced motion sickness (Graybiel et al. 1974; Keshavarz et al. 2015), and simulator sickness (Kennedy et al. 1993), which each result from exposure to different manners of novel sensory environments. Symptoms are wide ranging, including nausea, skin pallor, headache, disorientation, ocular discomfort, and, in extreme cases, vomiting (LaViola 2000). Although CS can be avoided entirely by simply terminating a VR session, some individuals experience severe and long-lasting nausea and discomfort following even brief exposures (Robillard and Bouchard 2007). This is an undesirable way for an individual to learn that they are susceptible to CS. Being able to predict motion sickness or CS based on some individual characteristics is therefore appealing, as it would allow these unpleasant experiences to be avoided. As such, there is a long history of discussion about the causes of motion sickness and related maladies. The dominant theory of motion sickness etiology emphasizes the role of accumulated conflicts between obtained and expected sensory cues in producing the nausea response (Oman 1990; Reason and Brand 1975; Thornton and Bonato 2013). This research has been extended by models and studies of motion sickness whose results suggest that the provocative stimulus for motion sickness may be a mismatch in the sensed and predicted vertical (Bles et al. 1998; Bos et al. 2008). Others have proposed a central role for postural instability in motion sickness (Riccio and Stoffregen 1991; Takada et al. 2007). There are significant challenges involved in falsifying these theories of motion sickness etiology, and experimental evidence often supports multiple theories (as discussed by, e.g., Ketelaar and Ellis 2000; Nooij et al. 2017; Stoffregen and Riccio 1991; Weech et al. 2018).

Although there are several hypotheses about the causes of CS symptoms during VR use, existing theories have yet to offer techniques that prevent its occurrence. The problem of CS requires a solution if society is to benefit from the potential impact of VR technology. The fact that large individual differences exist in terms of CS susceptibility suggests that some factors that differ between susceptible and nonsusceptible users can be identified and used to guide the development of CS prevention methods (such as tailoring content delivery based on individual susceptibility). Existing literature has identified several factors that may explain individual heterogeneity in CS, but most studies focus on one rather than multiple contributing factors. Here, we first provide an overview of the literature with a focus on highlighting evidence for a multifactorial causal structure for CS. We then describe an experiment in which we collected several measures of sensorimotor processing (e.g., balance control and self-motion sensitivity) before participants were exposed to VR and used these measures to construct a multiple-regression model with the aim of predicting the severity of CS.

### Balance Control

Recent research has suggested that individual differences in CS are related to balance control and self-motion perception (Dennison and D’Zmura 2017; Keshavarz et al. 2015; Riccio and Stoffregen 1991; Sadiq et al. 2017). Perceiving and controlling self-motion requires the integration of multisensory cues (e.g., vision, audition, proprioception, and vestibular sense) to derive knowledge about the state of the body in space. VR exposure can lead to a “sensory rearrangement” (Reason and Brand 1975; Welch 2002), where the learned relationships between sensory modalities are modified; for example, in VR, small but critical delays between sensory feedback across modalities can affect the perception and control of the temporal evolution of an action (Biocca et al. 2001). As well, visual and vestibular cues that convey information about the state of the head-on-body are frequently incongruent in VR, which may pose a challenge for the maintenance of stable postural control. The “postural instability theory” of sickness was formalized by Riccio and Stoffregen (1991; see also Chardonnet et al. 2017; Stoffregen and Riccio 1991; Stoffregen and Smart 1998; Takada et al. 2007), who suggested that motion sickness emerges as a consequence of postural instability resulting from unfamiliar environmental conditions. Support for this theory arises from studies showing that individuals with greater variability, velocity, and amplitude of head movements during quiet stance tend to report greater motion sickness severity when exposed to dynamic video games (Stoffregen et al. 2008). Other studies have documented similar findings when measuring center-of-pressure (COP) excursions, which are deterministically linked to head movements during quiet stance (Gatev et al. 1999). For instance, motion sickness produced by simple optic flow stimuli is predicted by the temporal dynamics of COP activity (Palmisano et al. 2018). Recent research has applied this theory in the context of CS, revealing that the area of postural sway tends to increase when participants experience CS (Chardonnet et al. 2017). Interestingly, although several of these studies have documented a positive correlation between postural sway and measures of motion sickness, other studies have found no evidence of this link (Dennison and D’Zmura 2018), whereas yet others have observed that individuals who experience stronger CS tend to demonstrate decreased postural sway (Dennison and D’Zmura 2017; Sadiq et al. 2017). These authors concluded that participants who experience CS may desire to remain stationary to avoid increased exposure to decoupled sensory streams in VR, resulting in reduced postural sway for individuals who are highly susceptible to CS (this phenomenon has been termed “VR lock”). Given the inconsistency between experimental results, there is a need for further examination of the relationship between balance control and CS.

### Visual Motion Perception and Vection

Consistent with the theory that CS arises because of stresses imposed on the sensorimotor control system by VR, recent evidence shows that individual variability in visual motion sensitivity may explain part of the heterogeneity in CS susceptibility rates (Allen et al. 2016). Individuals with a greater sensitivity to three-dimensional visual motion are more likely to opt for early termination during exposure to nauseogenic VR conditions and tend to experience higher levels of discomfort. Participants with greater visual sensitivity may have been more likely to detect the cue conflicts that occur during VR use, such as the visual-vestibular mismatch produced when self-motion is simulated with optic flow in VR. Notably, the stereoscopic three-dimensional motion stimulus presented by Allen et al. (2016) is highly relevant to the flow-parsing process that underpins self-motion perception, and as such, heterogeneity in susceptibility to visual self-motion illusions may explain why self-motion in VR results in sickness in some but not others (Keshavarz et al. 2015). It is possible that measures of vection (visually induced perception of self-motion; Dichgans and Brandt 1978) during visually simulated self-motion could reveal correlated differences in CS and visual self-motion sensitivity. Although some have characterized the effect of vection-inducing stimuli on vection ratings and postural sway (Berthoz et al. 1979; Palmisano et al. 2014) and shown that strong vection predicts high simulator and motion sickness (Hettinger and Riccio 1992; Hettinger et al. 1990; for a review, see Keshavarz et al. 2015), others have reported a negative relationship between vection and CS (Palmisano et al. 2017) or no relationship (Palmisano et al. 2018). The agreed consensus appears to be that the relationship is highly complex and requires further examination (Keshavarz et al. 2015; Palmisano et al. 2017) with more advanced analysis techniques.

### Vestibular Sensitivity

It is likely that differences in vestibulo-inertial perception play a key role in the variability observed in CS across individuals. Clinical research on patients with vestibular dysfunction has been important in this context, showing that individuals with vestibular labyrinth lesions do not exhibit sickness when exposed to a rotating visual field stimulus (Cheung et al. 1989, 1991; Johnson et al. 1999). When healthy participants are exposed to the same stimulus, symptoms that mirror those of CS result. The vestibular sense also plays a crucial role in the maintenance of balance control through detecting fluctuations in the inherently unstable human body (Peterka 2002). Given the relationship between postural instability and CS proposed by Riccio and Stoffregen (1991) and supported by others (e.g., Chardonnet et al. 2017; Takada et al. 2007), it is clear that vestibular sensitivity to self-motion is likely to modulate CS to some degree. Further support for this point arises from evidence of a strong comorbidity between vestibular migraine and motion sickness susceptibility (Money and Cheung 1983). Individual differences in vestibular functioning are also associated with increased susceptibility to motion sickness (Hoffer et al. 2003; Quarck et al. 1998, 2000) and sickness during spaceflight (Diamond and Markham 1991). For instance, Diamond and Markham (1991) found differences in vestibular-driven eye movements between astronauts who experienced sickness during spaceflight compared with those who did not. Other research has produced evidence of adaptation in the vestibulo-ocular reflex (VOR) following conditions that are often nauseogenic [e.g., free fall (Lackner and Graybiel 1981), off-vertical axis rotation (Tanguy et al. 2008; Young et al. 2003), and VR use (Draper 1998)]. Changes in the VOR during VR exposure may reflect vestibular habituation, driven by the visual-vestibular conflicts that accumulate during these provocative settings (Tanguy et al. 2008).

Physiological recordings in nonhuman animals have revealed strong evidence that neural pathways involving the vestibular nuclei underpin motion sickness (Balaban et al. 2014; Yates et al. 1998). These pathways are thought to be the same as those activated by ingestion of a toxin and involve the nucleus solitarius, lateral tegmental field, and parabrachial nucleus (Yates et al. 1998, 2014). Similarly, it has been proposed that sensory conflicts experienced during VR exposure are internally attributed to the ingestion of a toxin, thus triggering an emetic response (Treisman 1977). In support of a link between vestibular sensation and poison detection, complete vestibular labyrinthectomy is sufficient to extinguish the emetic response to many nauseogenic substances (Money and Cheung 1983). Although this sensory conflict theory is difficult to falsify, recent studies on the neurophysiology of the rhesus monkey have given insight into the neural basis of sensory conflict representation. Cullen and colleagues have revealed neuronal populations in the vestibular and cerebellar nuclei that show response profiles consistent with cancellation of “active” head movements but no cancellation of “passive” head movements (Brooks et al. 2015; Cullen 2014). The activity of these neurons is believed to represent the “sensory conflict” involved in motion sickness (Oman and Cullen 2014). Although this research presents a putative mechanism linking sensory conflict to motion sickness, there are several open questions regarding sensory conflict neurons (Oman and Cullen 2014). A common criticism of the sensory conflict account of CS is that is cannot be falsified because of the serious challenge of measuring sensory conflict in the brain (e.g., Ebenholtz et al. 1994; Stoffregen and Riccio 1991), and this recent progress in identifying sensory conflict neurons provides a significant step toward this goal.

Recent efforts to reduce visual-vestibular cue mismatch in VR support a partial vestibular basis for CS: Both galvanic vestibular stimulation (Cevette et al. 2012; Gálvez-García et al. 2015; Reed-Jones et al. 2007) and bone vibration applied near the vestibular organs (Weech et al. 2018) reduce the level of CS experienced during VR use. Visual-vestibular sensory conflict is also strongly implicated in the magnitude and latency of vection onset (Weech and Troje 2017; Wong and Frost 1981), which has a complex relationship with CS as discussed above. Despite the strong evidence that motion sickness appears to be partially attributable to high vestibular sensitivity, there are no studies to our knowledge that have investigated the relationship between vestibular self-motion sensitivity and CS.

### Additional Factors

Symptoms of CS and other forms of motion sickness are correlated with heightened autonomic nervous system activity (Golding 1992; Harm 2002; Ohyama et al. 2007). Common measures of CS emphasize qualitative experiences that indicate abnormal physiological functioning, such as excess sweating, burping, and stomach awareness (Kennedy et al. 1993). When exposed to nauseogenic conditions, marked differences in hormonal secretion (particularly vasopressin, but also adrenocorticotropin and growth hormone) have been observed for individuals who are susceptible to motion sickness (Eversmann et al. 1978; Kim et al. 1997; Kohl 1985). Individuals of Asian ethnicity, who demonstrate increased vasopressin release when exposed to provocative stimuli, are also more susceptible to motion sickness compared with European and African American individuals (Klosterhalfen et al. 2005; Stern et al. 1996). A robust association has been documented between phasic skin conductance measured at the forehead and both motion sickness (Golding 1992) and CS (Gavgani et al. 2017), whereas measures such as respiration rate and finger skin conductance were not associated with sickness severity. Other physiological measurements such as electroencephalography (EEG) and electrocardiography (ECG) demonstrate some predictive validity for self-reported CS scores (Dennison et al. 2016; Kim et al. 2005). However, these measures are typically obtained during VR exposure when CS symptoms have already emerged. The practical utility of predicting CS from online measurements is limited by the fact that CS symptoms can emerge quickly and can be long lasting, even upon exiting the nauseogenic conditions (Bos 2011; Kennedy et al. 2000; Regan 1995). Although it may be possible to gather physiological data before VR exposure and use those to predict the emergence of CS, we are not aware of any studies that have used such an approach.

The experience of motion sickness and CS may also depend on sex. In several studies a higher severity of sickness was reported by women compared with men (De Leo et al. 2014; Flanagan et al. 2005; Jaeger and Mourant 2001), although other research has failed to find any differences between sexes (Gamito et al. 2008; Knight and Arns 2006; Ling et al. 2013). The effects of sex have been attributed to differences in mental/spatial rotation during virtual environment exploration, which may modulate disorientation (Parsons et al. 2004). The discordant findings regarding sex differences in motion sickness might be related to changes in hormonal and sympathetic nervous system activity during the menstrual cycle (Golding et al. 2005).

### Approach of the Present Study

The studies discussed here provide a wealth of evidence about the possible causes of heterogeneity in CS. Principally, these factors include balance control (Chardonnet et al. 2017; Riccio and Stoffregen 1991; Takada et al. 2007), vestibular motion sensitivity (Diamond and Markham 1991; Hoffer et al. 2003), and visual motion/self-motion perception (Allen et al. 2016; Keshavarz et al. 2015). However, no studies have assessed the relative impact of each factor. Quantifying the role of each requires the assessment of responses in several behavioral tasks. These data, which are illustrative of the sensorimotor control system of a participant, may be used to construct a model that predicts the likelihood that the individual will experience CS, without exposing the individual to the discomfort of such an experience. This approach differs from several previous attempts to predict CS based on physiological or behavioral measures (e.g., Dennison et al. 2016; Kim et al. 2005; Nooij et al. 2017), which constructed predictive models using data that were collected during exposure to a nauseogenic stimulus.

The purpose of the present study was to characterize the degree to which CS susceptibility is attributed to individual differences in balance control and self-motion perception from visual and vestibular cues. Since a comprehensive evaluation of balance and sensory sensitivity would be impossible in a single session, our approach was to conduct measurements that we considered likely to relate to CS based on previously documented evidence, while acknowledging that there are other potentially important factors that we did not measure here. We predicted that measures of balance control would account for a large proportion of variability in CS scores, because of research suggesting that poor balance control precedes CS (Chardonnet et al. 2017; Takada et al. 2007). We also expected that susceptibility to vection would predict CS based on the association between vection and visually induced motion sickness (Keshavarz et al. 2015). Finally, motivated by literature that shows that increased vestibular sensitivity predicts high susceptibility to motion sickness (Diamond and Markham 1991; Hoffer et al. 2003), we expected that high vestibular self-motion sensitivity would be predictive of high CS scores. However, our main goal was to establish a multifactorial statistical model for predicting CS based on a combination of these factors.

## MATERIALS AND METHODS

### Participants

#### Recruitment.

Participants were recruited using mailing lists and posters on the University of Waterloo campus and were remunerated $10 per hour of participation. Participants were all naïve to the purpose of the experiment. This study was performed in accordance with the recommendations of Canada’s Tri-Council Policy Statement: Ethical Conduct for Research Involving Humans (TCPS2) by the University of Waterloo’s Human Research Ethics Committee with written informed consent from all subjects. All subjects gave written informed consent in accordance with the Declaration of Helsinki. The protocol was approved by the University of Waterloo’s Human Research Ethics Committee.

#### Demographics.

Thirty undergraduate and graduate students participated in the study [11 were male; mean (SD) age = 22.87 (3.94), age range = 18–30]. All participants had normal or corrected-to-normal vision and reported having no musculoskeletal, neurological, or balance disorders.

Participants were invited to optionally record their ethnicity, and 23 of the 30 participants chose to do so. Of those who reported ethnicity, 10 reported “Asian” and 13 reported “European.” In addition, participants were invited to record their daily activity level (low/moderate/high). In total, 7 reported “low” daily activity, 18 reported “moderate” activity, and the remaining 5 reported “high” daily activity.

### General Procedure

The general procedure of the experiment is described here, and further details are provided for each task in materials and methods, *Specific Procedures*.

Before commencing the experiment, participants were introduced to the purpose of the study through the letter of information and consent. Participants then completed a questionnaire to assess the incidence of motion sickness in their daily activities and in childhood [Motion Sickness Susceptibility Questionnaire (MSSQ); Golding 1998] and reported demographic information.

In the second part of the study, participants completed balance control and self-motion tasks in a block design that was counterbalanced across participants. In the balance control tasks, participants were guided through the process of assessing their balance using force plates in five different sensory conditions (outlined in detail in materials and methods, *Specific Procedures*, *Balance control task*). In one of the balance tasks, we presented participants with a radial optic flow stimulus and assessed their visually induced sway as an index of vection susceptibility. In the vestibular self-motion sensitivity task, participants were passively rotated in yaw while seated on a motion base and were asked to report their direction of rotation (left/right).

In the third part of the study, participants completed two VR tasks and reported the level of postexposure CS. The VR tasks were administered with a predetermined order that was counterbalanced across participants. This part of the study was completed last in the experimental sequence to avoid any possible effects of sickness on performance in the other tasks. The total duration of the experiment was ~150 min.

### Specific Procedures

#### Balance control task.

**Fig. 1.**
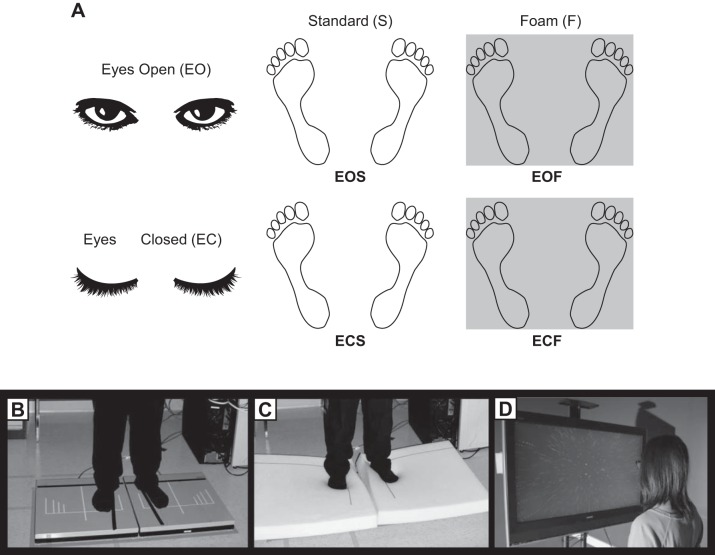
*A*: design of balance tasks: eyes-open standard (EOS), eyes-open foam (EOF), eyes-closed standard (ECS), and eyes-closed foam (ECF). *B*: depiction of standard stance on force plates (standard conditions). *C*: standing on foam that covered the force plates (foam conditions). *D*: observing a radial optic flow stimulus on a monitor (vection condition).

The balance control task comprised five sensory conditions in a block design where measures of postural sway were collected using two force plates (4060-05; Bertec, Columbus, OH) arranged in a side-by-side configuration, separated by ~1 cm. Prior to data collection, participants were asked to stand unshod in a standardized foot position (approximately shoulder width stance with toes rotated laterally by 14°; McIlroy and Maki 1997; see Fig. 1) with each foot on one of the two force plates. The outline of the feet was traced with markings to ensure consistent orientation of the feet across participants. After establishing the initial stance position, the participants were asked to stand quietly for 30 s with hands at their side in five sensory conditions that manipulated their visual or somatosensory inputs. Figure 1 depicts the conditions of the balance control task. The five sensory conditions were standing with eyes open [eyes-open standard (EOS)], standing with eyes closed [eyes-closed standard (ECS)], standing on foam with eyes open [eyes-open foam (EOF)], standing on foam with eyes closed [eyes-closed foam (ECF)], and standing while observing a radial optic flow stimulus that induced vection (V; see materials and methods, *Specific Procedures*, *Vection task*). In eyes-open conditions, the participants were asked to fixate on a cross (5 cm) placed at eye level on a wall 2.74 m in front of the participant (visual angle of the cross was approximately 1 × 1°). After each trial, participants were required to take a 10-s break. Trials in this task were blocked by sensory condition and administered in a predetermined randomized order. If the participant intentionally moved or stepped off the force plates during a trial, the trial was recollected. The task lasted ~45 min including setup of the apparatus and instructions.

Vertical ground reaction force (F*_z_*) and moments of force (*x* and *y* planes) from the force plates were recorded over a 30-s period for eight trial repetitions for each sensory condition. The force plate data were amplified online using an internal digital preamplifier, sampled at a rate of 1,000 Hz, and stored for off-line analysis. The force plates were calibrated before data collection for each participant. The force plate data were acquired using a custom-built LabVIEW program (National Instruments, Austin, TX). Our choice of trial duration (30 s) was motivated by evidence that this duration provides the optimum test-retest reliability (Le Clair and Riach 1996). In addition, measuring stance for 30 s is a common standard for postural sway measurement in adults and the clinical population because longer durations (1 min or more) may be too lengthy for a patient (Duarte and Freitas 2010).

Postprocessing of force plate data consisted of low-pass filtering (6-Hz, dual-pass 2nd-order Butterworth filter), calculation of COP in anteroposterior (AP) and mediolateral (ML) positions, and extraction of COP parameter (sway path length) using a custom-made LabVIEW program. Sway path length is defined as the total length of the COP path in 30 s, which is approximated by the sum of the distances between two consecutive points on the COP path (Hufschmidt et al. 1980; Prieto et al. 1996).

#### Vection task.

As part of the balance control section, participants observed a vection stimulus (V condition) while balance measures and verbal reports were collected. Participants observed a radially expanding optic flow stimulus that consisted of a cloud of 1,000 randomly positioned dots (0.25° visual angle; blue dots on a black background; a video depiction is provided in Supplemental Movie S1; Supplemental Material for this article is available online at the *Journal of Neurophysiology* website). The movement of the dots toward the observer was intended to give rise to the impression of linear translation of the observer in the AP axis. The dots contained linear perspective and relative size cues to depth. The visual stimulus included an oscillation component (0.5-Hz mediolateral frequency; 1-Hz ventrodorsal frequency), which is known to enhance the sense of vection (e.g., Apthorp and Palmisano 2014; Palmisano and Kim 2009).

The optic flow stimulus was presented on a liquid crystal display (LCD) screen (76 × 133 cm) that was adjusted to eye height and positioned 53 cm ahead of the observer (visual angle was approximately 71 × 103°). Before the block of vection trials, the experimenter explained the feeling of vection (“You may feel the illusion that your own body is moving through space”). The investigator provided the example that vection can occur when looking out of a window at a moving vehicle. Participants were required to verbally confirm that they understood what was meant by vection. Each participant was shown an example of the vection stimulus and asked whether they indicated vection. All participants except one reported vection (note that data from this participant were not excluded from analyses). Next, the experimenter instructed participants that they were required to indicate how strongly they felt vection after each trial on a scale of 0–10. The anchors provided were “0: no vection at all” to “10: the strongest possible feeling of vection.” Participants were positioned in front of the LCD screen while they stood on the force plates. Before each trial began, participants fixated on a central cross (~0.5°) on the LCD screen that specified where gaze should be located during the trial. The fixation cross disappeared once the vection stimulus began. The vection stimulus was presented for 30 s, during which balance control data were obtained from the force plates. Finally, the experimenter asked the participant to verbally report the strength of vection experienced during the trial on the 0–10 scale.

#### Vestibular direction estimation task.

We measured vestibular sensitivity to self-motion in terms of the ability of the participant to estimate the direction of yaw rotation on a motion platform when visual, auditory, and proprioceptive cues were minimized. Although there are a multitude of possible axes and rotation frequencies at which vestibular thresholds are commonly measured, we selected 1-Hz yaw rotations because of the high prevalence of yaw head movements in the natural environment, as well as the similarity between 1-Hz sinusoidal acceleration profiles and natural head movements (Demer et al. 1992). Conflicts between visual and vestibular yaw rotation cues are nauseogenic (e.g., Nooij et al. 2017), suggesting that yaw thresholds are relevant for studying CS. Vestibular yaw thresholds are also well studied, permitting comparisons between our results and others (e.g., Benson et al. 1989; Grabherr et al. 2008; Soyka et al. 2012).

**Fig. 2.**
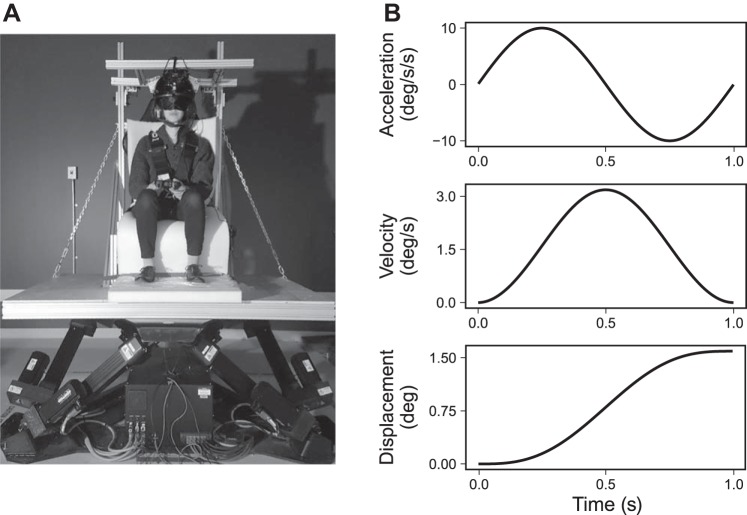
*A*: apparatus used in the vestibular self-motion sensitivity task. Participants were positioned on a cushioned seat mounted to a motion base. *B*: acceleration, velocity, and displacement profiles of an example movement of the platform (in this case, 1.6° angular displacement).

Participants were seated on a racing chair (A4; Corbeau, Sandy, UT) that was mounted to a motion base (MB-E-6DOF/12/1000KG; Moog, Elma, NY; Fig. 2*A*) with a custom-built frame. A five-point harness was used to ensure the participant’s position was stable, and the participant’s head was secured in place with a helmet. The vertical and horizontal location of the helmet was adjusted by the researcher to comfortably fit the head of each participant and to ensure that the axis of rotation of the motion platform intersected with the center of the participant’s head. Participants used a blindfold and earplugs and were exposed to white-noise auditory masking with active noise-canceling headphones while seated on the platform. Foam padding was mounted to the surface of the chair and the platform beneath the feet of participants to reduce the potential influence of proprioceptive cues. Participants were required to wear long sleeves and nitrile gloves to avoid an influence of air resistance on direction judgments. For the same reason, a fan mounted to the platform was directed at the face of the participant throughout the task.

In each trial, participants were rotated in yaw (either left or right) for 1 s. The rotation of the platform adhered to a sinusoidal angular acceleration profile (see Fig. 2*B*). As in previous studies (e.g., Grabherr et al. 2008; Soyka et al. 2012) we manipulated the peak velocity (°/s) of these motion profiles in each trial and characterized participants’ thresholds in terms of peak velocity values. We predetermined a list of 20 possible platform movements that each had a different peak velocity. The peak velocity values ranged between 0.05 and 6.0°/s in 20 increments that were spaced logarithmically. Similarly to previous research (e.g., Soyka et al. 2012), we used a staircase procedure where the motion profile was selected from the list on a trial-by-trial basis according to the previous responses of the participant (psi-marginal; Kontsevich and Tyler 1999). In brief, in each trial the adaptive procedure selects the stimulus intensity for which the participant’s response would be most informative for estimating the participant’s psychometric function [for additional details, see Kontsevich and Tyler (1999) and Prins (2013)]. We used the final estimate of the psychometric function from the adaptive procedure to derive an estimate of the 75% direction discrimination threshold for yaw rotation.

At the start of each trial, a beep was played to signal movement onset (440 Hz, 250 ms), and then the participant was rotated in yaw (left or right, 1 s). After the participant was moved, a second beep was presented (880 Hz, 250 ms) to indicate that the participant should input their response (i.e., in which direction they rotated: left or right). The response was inputted using a handheld gamepad (Logitech F310), where two buttons indicated “left” or “right.” The participant’s response was entered into the psi-marginal adaptive algorithm, which then selected an appropriate stimulus intensity for the subsequent trial. A third beep was played after the response had been inputted (220 Hz, 250 ms). After the response was made, the motion base rotated at a constant speed to its initial orientation in preparation for the next trial (the return movement was 7 s, corresponding to 0.14 Hz; the maximum possible velocity of the return movement was 0.85°/s, which is subthreshold at this frequency; Grabherr et al. 2008). A single practice trial at the start of the task consisted of the same motion trajectory for each participant (2.1°/s peak velocity).

The 150 trials of this task were separated into adaptive staircases for each direction (left/right) with 75 trials in each. Each trial required ~13 s to perform (0.25-s beep, 1-s rotation, 0.25-s beep, ~3-s response, 7-s return to center of rotation, and 1.5-s pause). The task lasted ~45 min including setup of the apparatus and instructions.

#### VR tasks.

**Fig. 3.**
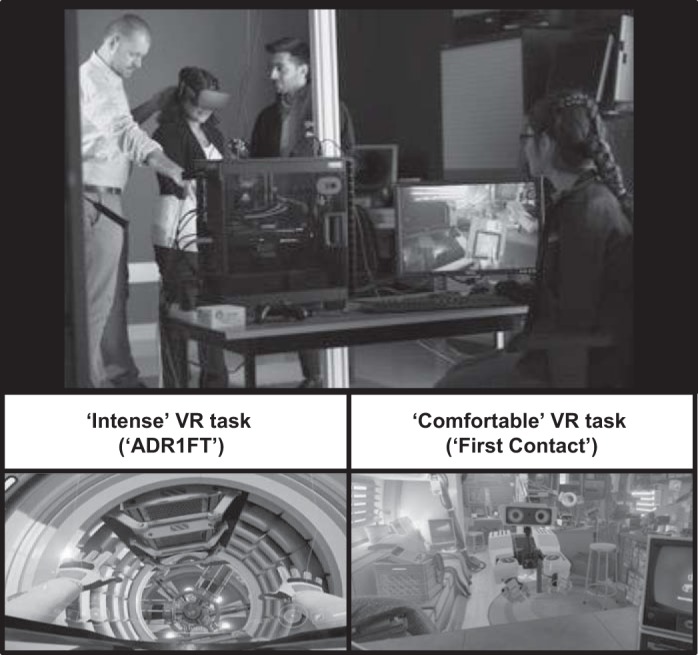
A participant is introduced to the environment in one of the two virtual reality (VR) tasks (*top*), which are depicted in the screenshots below.

Participants were guided through the process of fitting a head-mounted display (Rift CV1; Oculus VR, Menlo Park, CA) and adjusting the device (interocular distance and position on face) before completing the VR tasks. Participants were asked to play two types of VR content that have been rated on the Oculus Store comfort-rating system as either “intense” or “comfortable” (Fig. 3). The two VR tasks were completed sequentially in a predetermined order that was counterbalanced across participants. Each task lasted 7 min in total.

The first VR task was “ADR1FT,” in which participants played the role of an astronaut freely exploring a simulated space station. The participant navigated through the environment using a handheld gamepad (Xbox One Wireless Controller; Microsoft, Redmond, WA). This experience is rated as intense on the Oculus Store website (https://oculus.com/experiences/rift/905830242847405/).

The second VR task was “First Contact,” in which participants observed and communicated with a robot in a simulated environment that depicted the interior of a travel trailer. The participant was encouraged by the robot to perform simple actions such as picking up and throwing objects using six-degrees-of-freedom motion-tracked controllers (Touch Controllers; Oculus VR) that were held in the right and left hands. This experience is rated as comfortable on the Oculus Store website (https://oculus.com/experiences/rift/1217155751659625/).

After each VR task, participants completed the Simulator Sickness Questionnaire (SSQ; Kennedy et al. 1993): a checklist of 16 symptoms (e.g., nausea, headache, and sweating) to be rated on a scale of “none,” “slight,” “moderate,” or “severe.” The VR tasks lasted ~30 min including setup of the apparatus and instructions.

## RESULTS

### Descriptive Results

#### Balance control data.

Average sway path length measures across participants were normally distributed in each balance control condition (nonsignificant Kolmogorov-Smirnov tests, *D* ≤ 0.13, *P* ≥ 0.64). We observed no significant outliers in any condition (1-sample Dixon outlier tests, *Q* ≤ 0.34, *P* ≥ 0.093).

**Fig. 4.**
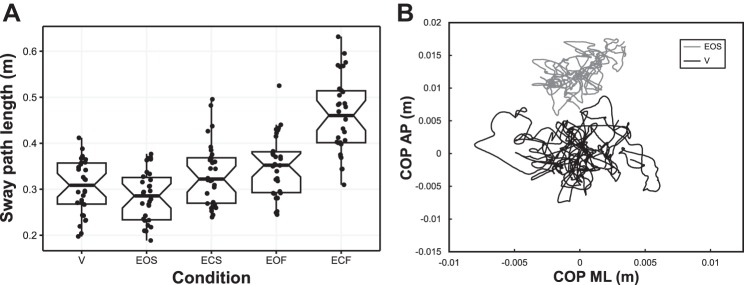
*A*: notched box plot depicting balance control measures for each condition. Thick horizontal lines are group medians. Black dots are participant averages. Note that although one data point for the eyes-open foam (EOF) condition was outside of 1.5 interquartile ranges, this was not a significant outlier (1-sample Dixon test, *Q* = 0.34, *P* = 0.093). *B*: example center-of-pressure (COP) traces from trials in the eyes-open standard (EOS) and vection (V) conditions. Traces depict COP excursions during a 30-s duration. AP, anteroposterior; ECF, eyes-closed foam; ECS, eyes-closed standard; ML, mediolateral.

Figure 4 depicts the total sway path length for each condition. To assess whether balance control differed between the conditions, we conducted a one-way repeated-measures analysis of variance with a Greenhouse-Geisser correction. We found a significant difference between the conditions [*F*(3.04,88.14) = 95.12, *P* < 0.001]. Next, we conducted planned paired-samples *t*-tests based on our expectation that closing the eyes, standing on foam, and experiencing vection would increase balance control variability. The vection condition resulted in significantly higher sway path length (0.306 ± 0.011 m, mean ± SE) than the EOS condition (0.284 ± 0.010 m), which was our baseline comparison condition [*t*(29) = 2.87, *P* = 0.007]. In addition, sway path length was lower in eyes-open compared with eyes-closed conditions, both standing on foam and in standard stance [*t*(29) ≥ 6.87, *P* < 0.001], and sway path length was lower in standard standing conditions compared with foam standing conditions in both eyes-open and eyes-closed visual conditions [*t*(29) ≥ 9.18, *P* < 0.001].

#### Vection strength data.

Participants’ estimates of the strength of vection experienced over eight trials were averaged to compute a single score for each participant. These data were normally distributed (nonsignificant Kolmogorov-Smirnov test, *D* = 0.13, *P* = 0.66). At the end of the block of vection trials we asked participants whether they experienced any sickness during the vection trials and received no affirmative responses.

Of the 30 participants we tested, 29 reported feeling vection (strength ratings across all participants: 3.30 ± 0.34). Rather than discarding the data of the participant who reported no vection (e.g., Riecke and Feuereissen 2012; Tarita-Nistor et al. 2008; Trutoiu et al. 2007), we retained their data for further analyses to avoid a possible sample bias.

#### Vestibular direction estimation data.

Direction discrimination reports (left or right) for each trial were used to update an adaptive algorithm for threshold estimation (psi-marginal; Kontsevich and Tyler 1999). Low threshold values indicate high sensitivity to self-motion and vice versa. We combined the left/right thresholds for each participant to obtain a single value representing the 75% threshold for estimating the direction of self-motion.

**Fig. 5.**
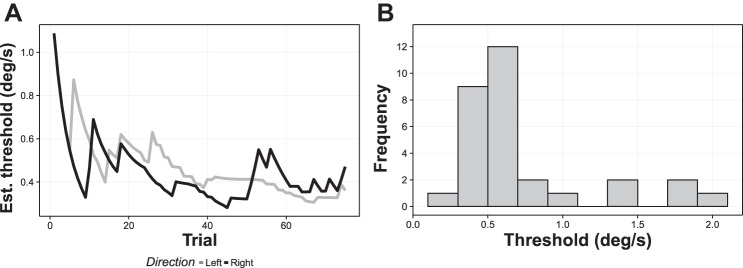
*A*: trial-by-trial estimates of the 75% threshold for a typical participant. Estimates from both the left and right staircases are plotted. The estimates start at a high magnitude and gradually approximate the participant’s true threshold over the series of trials. In this case, the final threshold value was ~0.4°/s. *B*: histogram of 75% direction discrimination thresholds across participants (bin width = 0.2°/s). Est., estimated.

Average yaw rotation thresholds were 0.74 ± 0.08°/s. Example data for a single participant are depicted in Fig. 5*A*. The vestibular threshold data obtained across participants were nonnormally distributed (significant Kolmogorov-Smirnov test, *D* = 0.27, *P* = 0.02; see Fig. 5*B*), so we applied a square root transform to the data (nonsignificant Kolmogorov-Smirnov test, *D* = 0.21, *P* = 0.11) before subjecting the data to further analysis.

#### CS data.

**Fig. 6.**
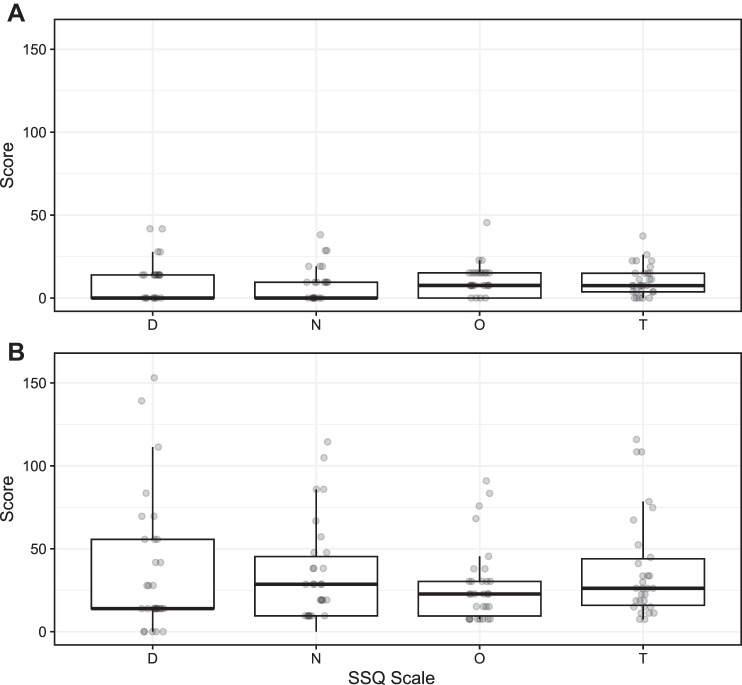
Box plots representing scores on the Simulator Sickness Questionnaires (SSQs) that were administered after the “comfortable” virtual reality task (*A*) and the “intense” virtual reality task (*B*): D, disorientation; N, nausea; O, oculomotor discomfort; T, total score. Thick horizontal lines are medians of each scale. Gray circles are participant scores.

We used participants’ responses on the SSQ to compute total scores for each VR task according to the formula of Kennedy et al. (1993). To verify that we were accurate in characterizing the two tasks as “intense” and “comfortable,” we compared the scores on each task and found a significant difference in sickness between the intense task (37.90 ± 5.93) and the comfortable task (10.22 ± 1.69), in the expected direction [Fig. 6; Welch’s *t*(33.69) = 4.49, *P* < 0.001].

We used the total scores on the SSQ for both VR tasks (intense and comfortable) to compute a difference score representing the effect of nauseogenic VR content on the participant’s comfort level, which we term “ΔCS scores.” The ΔCS scores were normally distributed (nonsignificant Kolmogorov-Smirnov test, *D* = 0.18, *P* = 0.28). Although other research has characterized CS as SSQ scores on a single VR task, we note that the ΔCS scores that we used here (i.e., difference in SSQs obtained after intense and comfortable VR content) were strongly correlated with SSQ total scores for the intense VR content [*r*(28) = 0.95, *P* < 0.001]. On the other hand, although the correlation between ΔCS scores and SSQ total scores for the comfortable VR content was also significant, the relationship was weaker [*r*(28) = 0.36, *P* = 0.049].

### Correlations and Between-Group Effects

**Fig. 7.**
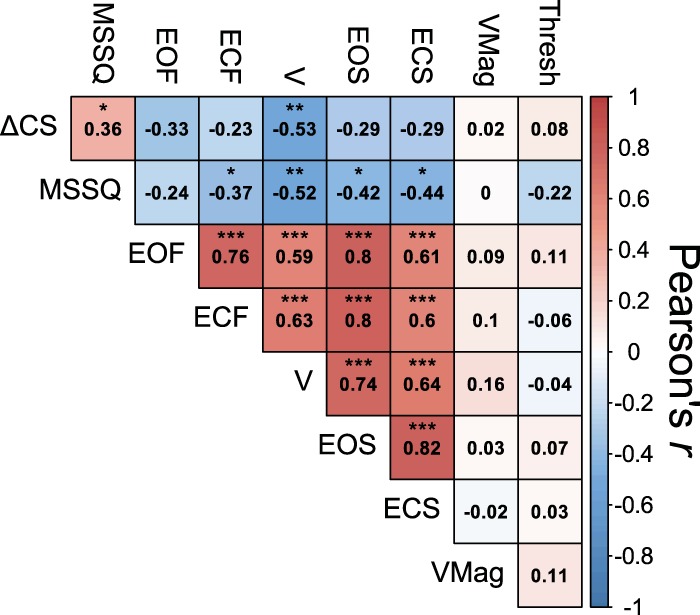
Correlation plot for measures obtained in the study. Correlations are Pearson *r*(28) values. ΔCS, cybersickness score; ECF, eyes-closed foam condition sway path length (SPL); ECS, eyes-closed standard condition SPL; EOF, eyes-open foam condition SPL; EOS, eyes-open standard condition SPL; MSSQ, Motion Sickness Susceptibility Questionnaire score; Thresh, vestibular threshold; V, vection condition SPL; VMag, vection strength rating. *P < 0.05, **P < 0.01, ***P < 0.001.

We conducted correlations to establish relationships between predictors and outcomes. A correlation plot of the data is depicted in Fig. 7. First, as expected, we identified a significant positive correlation between ΔCS and past motion sickness susceptibility [MSSQ; *r*(28) = 0.36, *P* = 0.048], suggesting that individuals who often experience motion sickness in provocative situations (e.g., boats and fairground rides) were also more likely to experience CS in virtual environments.

The balance control measures across the five sensory conditions were highly correlated. The Pearson *r* correlation values ranged from 0.59 to 0.82 (average value of 0.70). This suggests that the amount of sway demonstrated by a participant in one condition was predictive of the participant’s balance control in other sensory conditions, consistent with previous literature (Horak 1987; Winter et al. 1998, 2003).

**Fig. 8.**
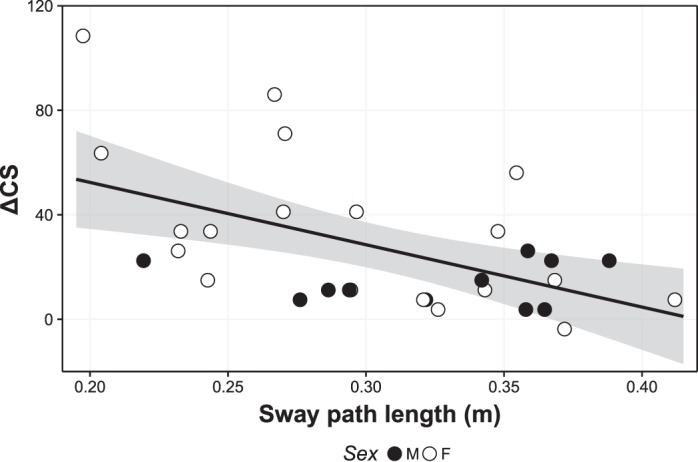
Scatterplot showing the relationship between average sway path length and cybersickness score (ΔCS) in balance control vection condition. Participant sex is indicated by shape fill (M, male; F, female). Shaded area depicts 95% confidence interval. Note that although there was one participant with higher ΔCS, it did not constitute a statistical outlier (Dixon 1-sided outlier test, *Q* = 0.36, *P* = 0.07) and removal of this data point would still have resulted in a significant negative correlation [*r*(27) = −0.43, *P* = 0.019].

We observed negative correlations between ΔCS and total sway path length in every balance control condition, with a mean Pearson *r* value of −0.33. Of these conditions, the only significant correlation between sway path length and ΔCS was in the vection condition [*r*(28) = −0.53, *P* = 0.002]. A scatterplot depicting this relationship is shown in Fig. 8. We also observed a significant correlation between sway path length in the vection condition and previous history of motion sickness susceptibility [MSSQ; *r*(28) = −0.52, *P* = 0.003].

To further inspect the relationship between ΔCS and balance control in the V condition, we computed seven other common balance measures from the V condition [ML and AP mean frequency, ML/AP root-mean-square error, mean radial displacement of the COP, and circular/rectangular sway path area (see Duarte and Freitas 2010)] and measured their relationship to ΔCS. The only significant correlation we observed was with AP mean frequency [*r*(28) = −0.43, *P* = 0.019; all other *P* ≥ 0.06], which was itself highly correlated with sway path length in the vection balance control condition [*r*(28) = 0.67, *P* < 0.001].

As an additional follow-up analysis, we examined whether sway in the V condition was correlated with each subscale of the SSQ: oculomotor discomfort, nausea, and disorientation (Kennedy et al. 1993). The ΔCS scores for each subscale (i.e., scores after the intense VR task minus scores after the comfortable VR task) were negatively correlated with each subscale [oculomotor: *r*(28) = −0.55, *P* = 0.002; nausea: *r*(28) = −0.47, *P* = 0.008; disorientation: *r*(28) = −0.43, *P* = 0.017]. Given the similarity of these correlations and since the SSQ total score was highly correlated with the individual subscale scores [oculomotor: *r*(28) = 0.89, nausea: *r*(28) = 0.91, disorientation: *r*(28) = 0.93], further analyses were performed only on ΔCS for the SSQ total scores.

With respect to participant sex, we found that ΔCS significantly differed between male and female participants [Kruskal-Wallis *χ*^2^(1) = 3.95, *P* = 0.047], with women experiencing more ΔCS (34.84 ± 6.97) than men (13.94 ± 2.48; Fig. 8). There was no difference between men and women with respect to MSSQ total scores [Kruskal-Wallis *χ*^2^(1) = 1.61, *P* = 0.204]. Additionally, we found no differences in total sway path length between men and women in any balance condition [Kruskal-Wallis tests, *χ*^2^(1) ≤ 1.84, *P* ≥ 0.175].

We obtained several nonsignificant tests for the effects of behavioral or demographic factors on CS. There was no correlation between ΔCS scores and participant age [*r*(28) = 0.20, *P* = 0.297], vestibular thresholds [*r*(28) = 0.06, *P* = 0.743], or average vection strength ratings [*r*(28) = 0.01, *P* = 0.964]. In addition, there was no effect of the self-reported daily activity level of the participant [Kruskal-Wallis *χ*^2^(2) = 1.05, *P* = 0.590] or ethnicity [Kruskal-Wallis *χ*^2^(1) = 0.88, *P* = 0.349] on ΔCS scores.

There were no significant correlations between vestibular thresholds and any other factor [*r*(28) ≤ 0.22] or between vection strength and other factors [*r*(28) ≤ 0.16].

### Principal Components Regression Analysis

To estimate the contribution of each candidate factor to CS, we constructed a multiple-regression model. However, our data set included a set of highly multicollinear variables, such as the sway measures obtained in the five balance control conditions. As well, MSSQ scores were significantly correlated with scores on some balance control conditions (see Fig. 7 above). This issue precludes a standard multiple-regression approach. Instead, we used principal components regression (PCR), which involves conducting a principal components analysis (PCA) on the data set and then subjecting the principal components (PCs) to a multiple-regression model. PCA is an unsupervised dimensionality reduction technique that results in uncorrelated linear combinations of variables (PCs) that are ordered by the amount of variance they explain in the original data set. This procedure eliminates multicollinearity at the expense of the interpretability of the predictors (Massy 1965).

**Fig. 9.**
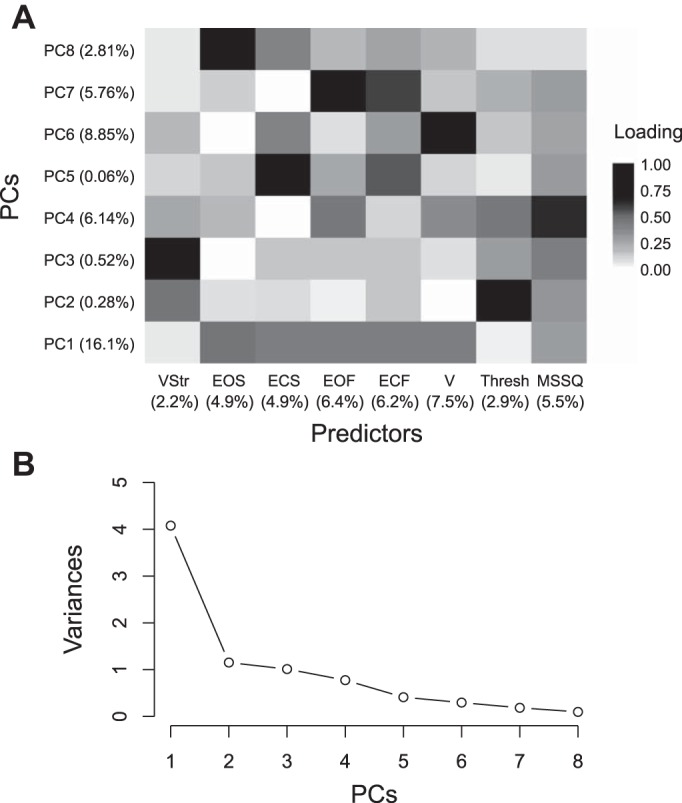
*A*: principal component (PC) loadings for each predictor. Percentage values on left indicate the amount of variance in cybersickness scores (ΔCS) accounted for by each PC; percentage values on the bottom indicate the amount of unique variance in ΔCS accounted for by each predictor. Darker shades depict higher loadings, representing a greater expression of that factor on the PC. ECF, eyes-closed foam condition sway path length (SPL); ECS, eyes-closed standard condition SPL; EOF, eyes-open foam condition SPL; EOS, eyes-open standard condition SPL; MSSQ, Motion Sickness Susceptibility Questionnaire score; Thresh, vestibular threshold; V, vection condition SPL; VStr, vection strength rating.* B*: scree plot showing variances in the initial data set accounted for by each PC (eigenvalues).

We identified eight factors to be used in the PCA. These factors were motion sickness susceptibility (MSSQ), vestibular thresholds, vection magnitudes, and total sway path length measures from the five balance conditions. In PCA, the first PC always explains the most variance, and in our data set, PC1 explained 50.96% of variance in the original data set, whereas PC2–4 and PC5–8 carried ~37% and 12% of the remaining variance, respectively (see Fig. 9 for a scree plot of PC variances). Whereas the PCs are less readily interpretable than the original factors, insight into what they represent can be gained by inspection of the factor loadings for each PC. Figure 9*A* depicts these factor loadings, where higher values indicate a greater expression of that factor in the PC. For instance, it can be determined from Fig. 9*A* that PC1 primarily represents a linear combination of all five balance control conditions and MSSQ scores. We also computed the percentage of variance in ΔCS scores uniquely explained by each predictor. This was achieved by multiplying a predictor’s loading for each PC by the variance in ΔCS explained by that PC. The largest amount of variance was explained by vection sway responses (7.5%), whereas vection strength responses accounted for the lowest percentage of variance in ΔCS among all the predictors we measured (2.2%).

We selected the components for the PCR model based on their correlation with ΔCS scores, with a predetermined criterion value of *r* = 0.20 (e.g., Dennison et al. 2016; Kim et al. 2005). PCs were entered into the model simultaneously to avoid the problems of stepwise regression techniques (Stephens et al. 2005). The PCs that met this criterion were PC1, PC4, PC6, and PC7 (Table 1). The results of the PCR revealed that the combination of these four orthogonal components significantly accounted for 36.8% of the variability in ΔCS scores [*R*^2^ = 0.37, adjusted *R*^2^ = 0.27, Cohen’s *f*^2^ = 0.59, *F*(4,25) = 3.64, *P* = 0.018]. Whereas PC6, PC4, and PC7 were not significant predictors alone (*P* ≥ 0.07), the first PC explained 16.1% of the variance in ΔCS scores, and this was revealed to be a significant component of the regression model (*P* = 0.018, see Table 1).

**Table 1.** Principal components regression parameters for predicting cybersickness scores

Predictor	β†	*t*	*P*	Partial *R*^2^
PC1	0.401	2.52	0.018*	0.161
PC6	−0.297	−1.87	0.073	0.088
PC4	0.248	1.56	0.132	0.061
PC7	−0.240	−1.51	0.144	0.058

PC, principal component.

* *P* < 0.05.

† β-Values are equivalent to Pearson *r* values in principal components regression.

**Fig. 10.**
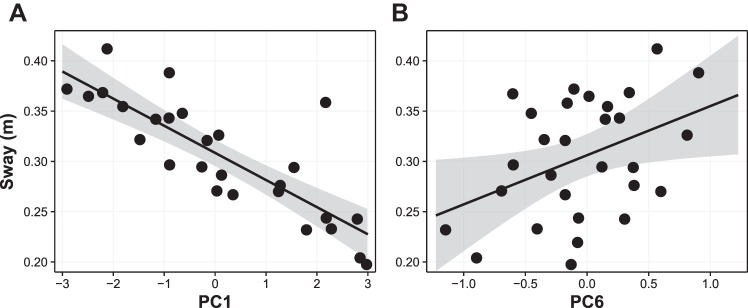
Scatterplot of total sway path length in the vection balance condition on the ordinate against scores on the first principle component (PC1, *A*) or PC6 (*B*) on the abscissa. Shaded areas are 95% confidence intervals.

Given that PC1 primarily represents a sign-reversed coding of sway values (e.g., see Fig. 10*A*), the sign of β for PC1 was positive. This reflects the negative correlation between ΔCS and the linear combination of balance control measures. On the other hand, for PC6 (which mainly expresses sway in the vection condition), the PC encodes high sway as positive and vice versa (Fig. 10*B*). Therefore, the negative β sign shows a negative relationship between sway and ΔCS. The same can be said for PC7 (primarily foam balance control conditions), where the negative β sign depicts the inverse relationship between total sway in these conditions and ΔCS scores that we reported previously. Although loadings on PC4 are spread more equally across predictors, the strongest loading is for MSSQ scores where the positive sign of β reflects the positive correlation between MSSQ and ΔCS scores.

### Model Comparison

We aimed to determine whether the model that we selected based on correlation between PCs and CS was the optimal model. An alternative would be that a saturated model (all PCs as predictors) predicted ΔCS significantly better or that an even more reduced model (PC1 alone) predicted ΔCS equally as well as our chosen model (and would therefore be considered “optimal” because of parsimony). We calculated the Akaike information criterion (AIC) for all models and adopted a ΔAIC value of 2 as a criterion for preferring the lower-AIC model. Results are shown in Table 2. The model based on correlations between PCs (PC1, PC4, PC6, and PC7) exhibited the best model fit.

**Table 2.** Akaike information criterion for multiple-regression models

Predictors	AIC	ΔAIC
PC1, PC4, PC6, and PC7	278.97	
All PCs (saturated)	285.17	−6.20
PC1	281.49	−2.52

AIC, Akaike information criterion; PC, principal component.

## DISCUSSION

Here we aimed to estimate the contributions of several candidates thought to play a role in CS, including vestibular thresholds, balance control, and vection susceptibility. Correlation analysis revealed a significant negative relationship between ΔCS and sway path length when participants observed a vection stimulus. We conducted a PCR to assess the contribution of orthogonal linear combinations of each candidate factor on ΔCS and interpreted the factor loadings for the significant predictors. We found strong evidence supporting the role of vection susceptibility and the role of balance control in CS. The correlation between balance control and ΔCS that we observed was negative, opposite to that reported in previous literature (e.g., Chardonnet et al. 2017; Stoffregen and Smart 1998; Takada et al. 2007), although a negative correlation has also been identified in other recent work (Dennison and D’Zmura 2017, 2018; Sadiq et al. 2017). We found no evidence of a link between ΔCS and vestibular thresholds or verbal ratings of vection strength. Our results demonstrate that behavioral and self-report data gathered before exposure to VR can be used to assess the likelihood of CS emerging on an individual basis.

Given that our aim was to provide broad insights into the influence of factors that might determine CS, there are several aspects of each of these factors that were not measured here. For instance, although we found no relationship between 1-Hz vestibular yaw thresholds and CS, we are of course unable to rule out relationships between CS and vestibular sensitivity at other frequencies or axes of rotation. For this reason, we believe our findings should form the basis for additional studies that can examine these sensorimotor indexes in more detail, for instance, by conducting multiple sessions of testing in a large sample of participants.

### Comparison with Previous Regression Studies

Previous work has adopted a regression approach to CS prediction. Kim et al. (2005) ran a stepwise regression analysis using several predictors including MSSQ scores and physiological data. A combination of ECG, EEG, and MSSQ scores predicted 46% of variability in CS. However, the model of Kim et al. was composed mostly of measurements taken during VR exposure (with the goal of CS classification), whereas each of the measures obtained in the present study were from preexposure assessments (with the goal of CS prediction). It is therefore not surprising that the variance explained by our model is lower than 46%. Indeed, the accuracy of our model is almost identical to that of a classification model produced by Dennison et al. (2016), who found that online measurements (during VR exposure) of bradygastric power, breathing rate, and blink rate obtained during VR exposure predicted 38% of variance in CS scores. In the present experiment our aim was to predict ΔCS using data that we obtained before VR exposure, but we anticipate that combining pretest and online physiological data would account for an even larger proportion of variance in CS.

Our approach shares some commonalities with a study by Nooij et al. (2017), who also combined several measures of sensorimotor processing, including vection strength and variability, head movements, and eye movement indexes (optokinetic nystagmus), in a predictive model of motion sickness. Similarly to previous work in this area (Dennison et al. 2016; Kim et al. 2005), the model was constructed using measures obtained during exposure to a nauseogenic stimulus (full-field visual yaw rotation). Their regression model composed of seven predictors accounted for nearly 80% of the variance in sickness scores (Fast Motion Sickness Scale; Keshavarz and Hecht 2011), with ~40% of that variance being attributable to mean overall vection strength. Importantly, their results showed that vection was only predictive of motion sickness within subjects (i.e., across multiple vection-inducing conditions) and not between subjects. In our study, vection strength ratings were also highly uninformative regarding an individual’s CS susceptibility. However, a distinction should be made between vection susceptibility during a nauseogenic stimulus (which we did not measure here) and vection susceptibility obtained before exposure to a stimulus that provokes sickness (which we found to be inversely related to CS). As a result, only limited comparisons between our study and previous experiments such as that of Nooij et al. (2017) can be drawn.

Although the regression approach has produced successful findings, other computational techniques may prove more powerful with respect to prediction. Whereas a recent study reported high classification accuracy using linear discriminant analysis with physiological data to categorize whether participants were viewing visual stimuli on a monitor or with a VR display (Dennison et al. 2016), future efforts should employ techniques such as linear discriminant analysis or support vector machines to construct predictive models of CS derived from behavioral and physiological measures.

### Balance Control

The theory that postural instability precedes motion sickness (Riccio and Stoffregen 1991) has proven extremely influential in research on CS, and the predictions of the theory have been supported several times (e.g., Chardonnet et al. 2017; Stoffregen and Smart 1998; Takada et al. 2007). Our results suggest that individual differences in balance control can predict tolerance of nauseogenic stimuli, but the direction of the relationship we identified between sway path length and ΔCS was negative. Our results agree with the findings of a separate body of literature wherein a null or negative association between sway and CS was found (e.g., Dennison and D’Zmura 2017, 2018; Sadiq et al. 2017). For instance, it has been revealed that postural instability caused by visual perturbations does not produce CS as predicted by the postural instability account of motion sickness (Dennison and D’Zmura 2018). Dennison and D’Zmura (2017) also showed that participants who exhibited less postural sway than others were more likely to experience CS. Those authors concluded that participants tended to reduce their body motion if they experienced CS, implicating CS as the cause of lower body sway, rather than vice versa. Our present results rule out this VR lock explanation given that we measured balance control before exposure to VR content and obtained negative correlations in all five conditions (although most trends were nonsignificant). However, we contend that our results do not directly contradict the postural instability theory of Riccio and Stoffregen (1991), given that increased sway may not be a good indicator of “instability” (Stoffregen et al. 2008). The adoption of a rigid, stationary posture does not necessarily reflect the readiness to respond to changing conditions that underpins dynamic stability (Błaszczyk 2008; Cho et al. 2014; Palmisano et al. 2018). Increased postural sway could demonstrate a more flexible balance control system and a readiness to adapt to novel sensorimotor conditions such as those presented by VR. A control strategy where the individual struggles to avoid postural adjustments in response to a compelling visual stimulus may minimize body sway. At the same time, this may constitute precisely the type of ineffective strategy that Riccio and Stoffregen (1991) identify as a precursor to motion sickness. As such, the present results complement the idea that balance control measures are valuable predictors of susceptibility to CS, although whether high-CS participants were “more stable” or “less stable” than others is open to interpretation.

Although it is possible that the difference between our results and other research can be attributed to methodological differences, the method we used to record balance control (30 s of standing in quiet stance before VR exposure) was similar to other research. For instance, Chardonnet et al. (2017) recorded balance for 30 s preexposure and postexposure to VR and used these data to calculate sway area. Palmisano et al. (2018) measured vection for 30 s and, in a separate task, they measured balance control during quiet stance for 60 s. The balance data were used to conduct recurrence quantification analysis, a nonlinear measure for balance control. Other studies are unclear with respect to trial durations (e.g., Palmisano et al. 2017). In addition, although our measure of CS (ΔCS) is different from that of most other studies, because of the high correlation between ΔCS and SSQ scores for the nauseogenic VR content used in our experiment (*r* = 0.95) we do not consider it likely that this accounts for the difference in results.

Since we measured a large number of variables, including balance control across five sensory conditions, vestibular thresholds, and motion sickness susceptibility, we elected a priori to extract only one measurement of balance control, namely, sway path length. Sway path length is a popular measure of balance control in research and has been used in several previous studies of motion sickness (e.g., Lubeck et al. 2015; Palmisano et al. 2014; Shahal et al. 2016). There are, however, numerous balance control measures that we did not assess here, the analysis of which would have significantly increased the number of statistical tests conducted at the possible cost of an increased false positive count. Although we measured the relationship between CS and a small number of other balance parameters (mean COP displacement, AP/ML mean frequency, and circular and rectangular area of displacement), only one of these balance control measures, AP mean frequency, was correlated with CS, suggesting that fore-aft sway in response to vection could offer an indicator for CS susceptibility. It should be noted, however, that the correlation between CS and sway path length was stronger than that between CS and AP mean frequency, supporting our choice to use sway path length as the primary dependent variable for balance control. Our results reinforce the idea that linear balance control measures such as sway path length are relevant to CS susceptibility. At the same time, other nonlinear measures (e.g., recurrence quantification analysis) should also be considered in future studies (note that our procedure here was not designed to permit recurrence quantification analysis), given that they demonstrate perhaps greater predictive validity for CS than traditional linear measures (Palmisano et al. 2018).

### Vection

Although some have proposed that vection plays a strong role in motion sickness (Keshavarz et al. 2015), some previous research has been unable to identify a strong relationship between vection and visually induced motion sickness (Palmisano et al. 2018). Our results extend this research by showing that vection balance responses predict CS in a nauseogenic virtual environment. We found that the sway in response to vection stimuli had the strongest predictive power for CS among all measures collected here. This result presents the future possibility of using sway responses to vection as a simple predictive tool for individual susceptibility to CS while avoiding nauseogenic conditions entirely (we note, however, that similar vection stimuli can produce motion sickness in wide field-of-view conditions; Palmisano et al. 2018, 2017).

What is the mechanism through which vection and CS are related? Although our results do not provide a direct answer to this question, one possible explanation is that the extent or magnitude of sensory conflict experienced by participants in VR also relates to the experience of vection. Large sway in response to vection indicates that visual cues are weighted higher than vestibular information, indicating a fast resolution of the visual-vestibular conflict experienced while a self-motion stimulus is presented. A link between sensory conflict resolution and CS habituation has been discussed recently (Gallagher and Ferrè 2018), and experimental results suggest that reducing sensory conflict may reduce CS (Reed-Jones et al. 2007; Weech et al. 2018) and facilitate vection (Weech and Troje 2017). With respect to neurophysiology, rapid cue conflict resolution is expected to produce diminished activity in sensory conflict neurons in the brain stem (although the existence of these cells in the human brain is an open question; Oman and Cullen 2014). Although it is feasible that reduced activity in this population of cells might be observed during conditions of sensory conflict for vection-susceptible individuals, this is an untested speculation at this stage. Future neurophysiology studies will likely be needed to test this prediction.

Whereas others have identified a positive association between vection strength and motion sickness (Hettinger and Riccio 1992; Hettinger et al. 1990), our results complement other work that has identified a negative relationship between CS and vection susceptibility as measured by magnitude ratings (Palmisano et al. 2017). Although Palmisano et al. suggested that the negative relationship they found was an artifact of their experimental design, our results constitute a replication of this effect in different settings, suggesting that the relationship may be a reliable one.

We expected to find significant agreement between COP parameters during vection and “subjective” measures of vection strength. In fact, there was a large degree of dissociation between these two ways of measuring vection. Although behavioral correlates are rarely considered in computational models of vection (Jürgens et al. 2016; Seno et al. 2017), this result highlights the dissociation between the behavioral and subjective components of the illusion. As shown in previous studies, the association between subjective and behavioral aspects of vection depends on the visual display conditions with respect to factors such as visual eccentricity and foreground-versus-background interpretation (e.g., Delorme and Martin 1986; Kawakita et al. 2000). Delorme and Martin (1986) highlight the frequent occurrence of cases where participants had synchronous postural reactions to optic flow stimulation without reporting any subjective vection responses. Other research also shows a weak relationship between sway and vection strength when vection is induced by a stimulus with viewpoint oscillation (Palmisano et al. 2014). We chose to present an oscillating stimulus because of the tendency for stronger and more reliable vection illusions with this type of stimulus compared with smooth vection (Apthorp and Palmisano 2014; Palmisano and Kim 2009). Therefore, the findings of the present study may have a limited applicability in other settings where different stimuli are used to evoke vection. Another point of consideration is that participants in the present study did not experience very strong vection overall based on subjective strength ratings. Future replications with more compelling stimuli are desirable. However, there was significantly greater sway path length observed in the vection condition compared with the EOS condition, reflecting that the optic flow stimulus used was sufficient for inducing vection.

### Vestibular Sensitivity

The vestibular threshold data we obtained were broadly in line with the results of others who have assessed the thresholds for small yaw rotations at similar frequencies (Grabherr et al. 2008). The most common thresholds in our experiment fell in the range between 0.5 and 0.7°/s, which closely aligns with the Grabherr et al. (2008) average threshold of 0.64°/s at 1 Hz. On the other hand, the thresholds we obtained were lower than those measured by Benson et al. (1989), who identified ~1°/s thresholds at 1.1-Hz rotation, and others reviewed by Soyka et al. (2012), although the difference may be attributed to methodological variations between the experiments. Notably, we did not find any relationship between vestibular thresholds and CS as predicted by research on vestibular dysfunction patients (Cheung et al. 1989, 1991; Johnson et al. 1999) and vestibular migraine patients (Money and Cheung 1983). An important qualification to our conclusion is that our measurements of vestibular thresholds were limited to the yaw axis because of experiment duration constraints. Other assessments, such as baseline measurements of VOR gain (Draper 1998; Tanguy et al. 2008; Young et al. 2003) or a “vestibulogram” of thresholds at different rotation frequencies (Grabherr et al. 2008), may offer more value for predicting CS than the measures we obtained here. Future studies that conduct a more comprehensive vestibular assessment are required to determine the optimal measures of vestibular functioning for predicting CS.

### Other Candidate Factors

Notably, although our regression model significantly predicted CS, there remained ~63% of variability in CS that was unexplained by the combination of our predictors. There are several candidates related to sensorimotor processing that merit exploration in the search for higher variance explained. These include genetic factors, the contribution of which should be assessed with a large sample. Several genes have recently been identified as linked to motion sickness in a full genome-sequencing study (Hromatka et al. 2015), but a genome-wide approach to CS susceptibility would not be possible barring a large proportion of the population undertaking a standardized VR experience. However, it appears likely that genetic factors modulate the sensorimotor indexes measured in our experiment, and future high-power studies will be needed to investigate the extent to which genetic polymorphism influences CS.

Although we did not include participant sex in our predictive model because of unequal numbers of male and female participants, our results support previous research that indicates that women tend to experience greater motion sickness severity than men (De Leo et al. 2014; Flanagan et al. 2005; Jaeger and Mourant 2001; although cf. Gamito et al. 2008; Knight and Arns 2006; Ling et al. 2013). Including the phase of a participant’s menstrual cycle in a regression model may also add predictive value (Golding et al. 2005), although we did not collect those data here. Conversely, our results did not provide evidence that CS differs as a function of ethnicity, which has been shown previously (Klosterhalfen et al. 2005; Stern et al. 1996).

Other promising latent factors include cue weightings across modalities, which may modulate the likelihood of detecting conflicts between sensory input and predictions (Oman 1990), between multisensory cues (Reason and Brand 1975), or between multiple estimates of the true vertical (Bles et al. 1998), which have all been implicated in the etiology of motion sickness. The relationship between CS and an individual’s ability to rapidly reweight multisensory cues in conditions of mismatch is also understudied (for a recent review, see Gallagher and Ferrè 2018). Although some authors have assessed rates of multisensory reweighting and measured their association with carsickness (Balter et al. 2004; note that no relationship was observed), there is a need to examine the reweighting function in VR conditions. In addition, an individual’s tendency to bind near-synchronous multisensory cues (the “temporal binding window,” Dixon and Spitz 1980; Wallace and Stevenson 2014) may predict the likelihood of sickness emerging in environments where a barrage of multimodal cues with varying latencies must be integrated by the central nervous system.

### Conclusion

In the future, the ability to predict susceptibility to CS based on a minimal set of measurements will enable the development of individualized recommendations for VR use, thus helping to prevent the nausea and discomfort that can occur rapidly and persist for several hours after VR use. Here we have shown that a combination of factors measured before experiencing intense VR content holds significant predictive power for the amount of CS that an individual experiences. The results indicate that the more a participant swayed in response to vection stimuli, the less CS they were likely to experience. Although our predictive model for CS depicts a central role for the destabilizing effect of vection on postural stability, we also found utility in other measures, such as balance control while standing on foam and self-reports of motion sickness susceptibility. Although our data do not directly rule out the involvement of any single factor in CS, we propose that differences in sensory reweighting may explain the relationship between vection and CS observed here. These results are intended to guide the development of future efforts to predict CS before its experience. Although it is clear that the measurements taken here could not be feasibly collected in a consumer setting, we contend that simplified methods of collecting the measures identified as important here (particularly vection sway responses) should be developed and rigorously tested in future studies with respect to their relationship to CS.

## GRANTS

This research was supported by an Ontario Research Fund grant and Canadian Foundation for Innovation John R. Evans Leaders Fund Grant 32618 to M. Barnett-Cowan and Natural Sciences and Engineering Research Council of Canada Grant RGPIN-05435-2014.

## DISCLOSURES

This research was supported by a grant to M. Barnett-Cowan from Oculus Research. The industry sponsor had no influence in the design or execution of the present research. No conflicts of interest, financial or otherwise, are declared by the authors.

## AUTHOR CONTRIBUTIONS

S.W., J.P.V., and M.B.-C. conceived and designed research; S.W. and J.P.V. performed experiments; S.W. and J.P.V. analyzed data; S.W., J.P.V., and M.B.-C. interpreted results of experiments; S.W. prepared figures; S.W. drafted manuscript; S.W., J.P.V., and M.B.-C. edited and revised manuscript; S.W., J.P.V., and M.B.-C. approved final version of manuscript.

## ENDNOTE

At the request of the authors, readers are herein alerted to the fact that a link to the data set described in this article is hosted on the Open Science Framework and can be accessed at the following location: https://osf.io/xusz2/. These materials are not a part of this manuscript and have not undergone peer review by the American Physiological Society (APS). APS and the journal editors take no responsibility for these materials, for the Web site address, or for any links to or from it.
